# Adrenocortical adenoma in a Sudanese girl with Beckwith-Wiedemann syndrome

**DOI:** 10.1186/s13633-019-0068-7

**Published:** 2019-11-22

**Authors:** Eman Abdalla Ali Elnaw, Awad Rhmattalla Abdalla, Mohamed Ahmed Abdullah

**Affiliations:** 1 Endocrine Division, Department of Paediatrics and Child Health, Faculty of Medicine, University of Khartoum, P.O.Box:102, Khartoum, Sudan; 2 Department of Surgery, Faculty of Medicine, University of Khartoum, Khartoum, Sudan

**Keywords:** Beckwith-Wiedemann, Adrenocortical adenoma, Virilization, Resource-limited setting

## Abstract

**Background:**

We report a case of right adrenocortical adenoma in a girl with features suggestive of Beckwith Wiedemann syndrome to show the importance of tumor surveillance in patients with Beckwith Wiedemann syndrome.

**Case presentation:**

A 4-years-old female with features suggestive of Beckwith-Wiedemann syndrome presented with 9 months history of virilization. Hormonal investigations results showed high levels of testosterone (2.3 ng/ml, normal values 0.1–0.4 ng/ml), and DHEAS (73 ng/ml normal values 1-6 ng/ml) with normal cortisol level. Computed tomography revealed a right adrenal mass. She underwent right adrenalectomy. Histopathological examination of the resected adrenal gland showed adrenocortical adenoma. Her postoperative evaluation showed a normal testosterone level.

**Conclusion:**

Adrenocortical neoplasms though rare in children are well documented in Beckwith-Wiedemann syndrome patients. So tumor surveillance protocol should be employed, even in a resource-limited setting for early tumor detection and a better outcome.

## Background

Beckwith-Wiedemann syndrome (BWS) is a pediatric overgrowth disorder involving a predisposition to tumor development (BWS, OMIM 130650). It has an estimated population incidence of 1 in 13,700. It results from several genetic mechanisms that lead to abnormal regulation of imprinted genes on chromosome 11p15. Most of the cases of BWS are sporadic while 15% of cases are familial showing an autosomal dominant mode of inheritance with incomplete penetrance [1]. It has a highly variable clinical presentation with abnormal growth being a common feature. This abnormal growth can result in macrosomia, hemihypertrophy or enlarged tongue. Abdominal wall defects, visceromegaly, and ear lobe creases are also known manifestations.

A wide range of both malignant and benign neoplasms have been reported in Beckwith-Wiedemann syndrome. Wilm’s tumor is the most common, followed by hepatoblastoma and adrenocortical carcinoma [2]. Some benign tumors have also been frequently observed in BWS of which adrenal adenoma constitutes 8% [2].

We report a case of virilizing adrenal adenoma in a 4 years old female with Beckwith-Wiedemann syndrome to emphasize the importance of tumor surveillance in BWS patients’ even in a resource-limited country like Sudan.

## Case presentation

Four years 3 months old female presented with 9 months- history of pubic hair growth and enlargement of the clitoris. Her condition was associated with adult-type body odor and aggressive behavioral changes. She had no axillary hair growth, acne, breast enlargement or vaginal bleeding, and no rapid weight or height gain.

She was born at 38 weeks gestation, following an uncomplicated pregnancy with no polyhydramnios. Her birth weight was 4.5 kg. She had been noted to have macroglossia with no feeding or respiratory difficulty. She was also noted to have right-sided hemihypertrophy involving the trunk and limbs. She had neither hypoglycemic attacks nor omphalocele. No documented follow up growth data were available. There was no family history of note and no consanguinity. A clinical diagnosis of Beckwith-Wiedemann syndrome was established, and her family had been reassured without a plan for further follow-up.

Examination showed a well-looking female with whole right side hemihypertrophy, protruded tongue and transverse ear lobe crease. Her height was 102 cm (50th centile) and weight 15 kg (25th centile) using the centers for disease control and prevention growth chart. She had normal blood pressure. Her Tanner staging was A_1_, P_2_, and B_1._ She had a prominent right labia, and clitoromegaly (1.5 cm). She had no hirsutism, acne or a palpable abdominal mass.

**Fig. 1:**
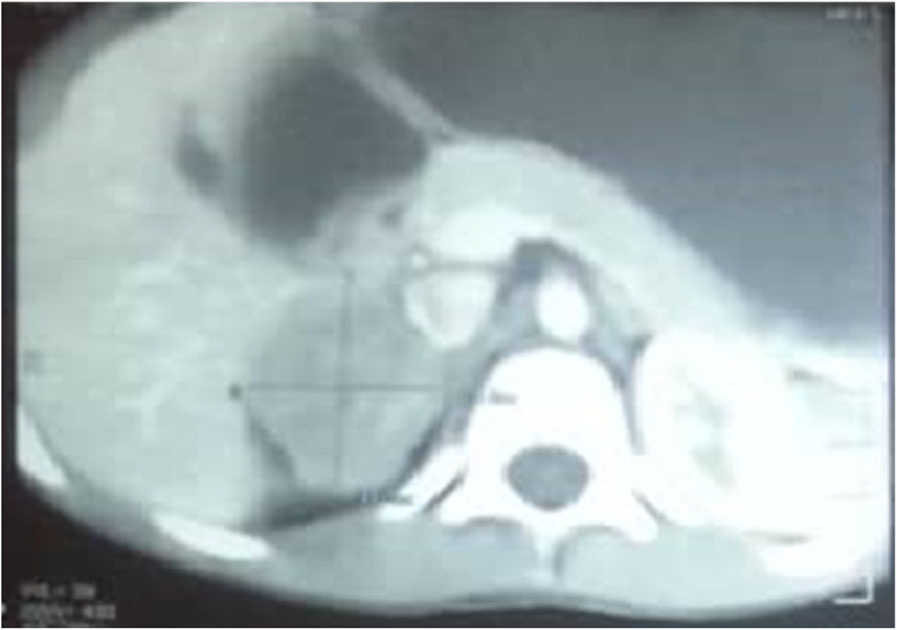
CT scan of the abdomen showing a right adrenal tumor

Left wrist x-ray showed a bone age of 7 years. An ultrasound scan showed a 2 cm well defined rounded hypoechoic lesion with multiple tiny calcifications in the right suprarenal region. Computed tomography (CT) showed a 37 × 32 mm well-circumscribed solid mass in the right suprarenal region (Fig. 1), no other abnormalities were found.

Hormonal investigation measured using fluorometric enzyme immunoassay showed elevated levels of testosterone 2.3 ng/ml. (0.1–0.4 ng/ml), progesterone 4.99 ng/ml. (0.1–0.3 ng/ml) and dehydroepiandrosterone sulphate 73 ng/ml. (1–6 ng/ml) with normal early morning cortisol level 15.6 ng/ml. (7–28 ng/ml) (Table 1).

**Table 1:** Hormonal profile of the patient before and after adrenalectomy

	Pre-operative Levels	Post-operative levels
Testosterone ng/ml (0.1–0.4)	2.4	0.02
Cortisol ng/ml (7–28)	15.6	1.9

During laparotomy, an enlarged right adrenal gland has been removed; measuring 5 × 3.5 × 3 cm and weighing 15 g, with an outer glistening nodular surface. The left adrenal gland was of normal appearance and was not biopsied.

Histopathology revealed well-circumscribed tumor-forming nests with trabeculae and sheets of polygonal cells with eosinophilic and vacuolated cytoplasm, fibrous bands with dystrophic calcification were seen. The tumor was not infiltrating the capsule, with no mitosis, atypia or necrosis, (Wieneke index score = 0). Findings were consistent with adrenocortical adenoma.

Convalescence was uneventful. The child has been covered during the perioperative period with a course of corticosteroids. One week post-surgery steroids have been stopped for 48 h and early morning cortisol has been evaluated. It was found to be low (1.9 ng/ml; normal values, 7–28 ng/ml). Thus the patient was started on oral maintenance steroid therapy, which will be continued until recovery of her adrenal function. The postoperative testosterone level was found to be 0.02 ng/ml. (0.1–0.4 ng/ml) (Table 1).

## Discussion

As BWS has a highly variable clinical presentation, major and minor diagnostic criteria have been set. Clinical diagnosis of BWS can be proposed based on the presence of three major criteria or two major and one minor criterion. In patients with subtle clinical features, molecular tests can be used to confirm the diagnosis [1]. Though no molecular test has been done in our case, a clinical diagnosis has been proposed based on the presence of three major criteria. These criteria were macroglossia, ear lobe creases, and right side hemihyperplasia.

Due to the high risk of neoplasia in BWS, many screening protocols have been suggested. An example of these suggested protocols is the one proposed by David J Amor and his colleagues. It includes serum alpha-fetoprotein measurement every 3 months until the age of 4 years, abdominal ultrasound focusing on the liver, kidneys, and adrenals every 3 months until the age of 8 years [3]. Though a 3 monthly scan is a feasible option in our setting, no single screening test had been done to our patient.

An adrenocortical neoplasm is rare in children and constitutes only less than 0.5% of all childhood tumors [4], but is well documented with BWS. Adrenocortical carcinoma represents 7% of the malignancies, while adrenocortical adenoma represents 8% of benign tumors reported in BWS [2].

This increased risk of adrenal neoplasm has been related to the over-expression of insulin-like growth factor 2 (IGF2) in BWS. Increased expression of this major growth regulator has also been linked to abnormal fetal adrenal development [5].

An adrenocortical neoplasm can be functional or nonfunctional. Functional tumors can present with virilization, Cushing syndrome or hyperaldosteronism, whereas nonfunctional tumors are detected incidentally. It has been noted that virilization was the most common clinical presentation of adrenocortical tumors in BWS patients [6]. Another observation is that unilateral androgen-secreting tumors can recur in the contralateral adrenal gland years after the first diagnosis [4]. This emphasizes the need for close follow up of these patients.

Though all hormone-producing adrenocortical masses need to be surgically resected, a pre-operative distinction of carcinoma vs. adenoma could help direct surgical management. Imaging using CT or magnetic resonance imaging can help in this discrimination, as adrenal carcinoma appears larger, more heterogeneous with calcification when compared to adrenal adenoma [7].

Histopathological scoring systems can also help to predict the malignant potential of a tumor. The Weiss score which is proposed to differentiate ACC from adrenocortical adenoma in adults has low accuracy in children. So it is replaced by Wieneke Index which is more accurate in this group [8].

Of the reported cases of adrenal neoplasm in BWS patients, Mizota reported bilateral asynchronous androgen-secreting adrenal adenoma in a girl with BWS [4]. Beauloye et al. have also reported a girl with a recurrence of androgen-secreting tumor 3 and half years after her first adrenalectomy [6]. These reports emphasize the need for regular follow up following adrenalectomy for early detection of possible recurrence.

## Conclusion

A wide range of both malignant and benign neoplasms has been reported in Beckwith-Wiedemann syndrome. Adrenocortical neoplasms though rare in children but are well documented in Beckwith-Wiedemann syndrome patients. So tumor surveillance protocol should be employed, even in a resource-limited setting for early tumor detection and a better outcome.

## Data Availability

Not applicable.

## Abbreviations

ACC: Adrenocortical carcinoma
BWS: Beckwith-Wiedemann Syndrome
CT: Computed tomography
IGF2: Insulin like growth factor 2
